# Splenic sarcoid reaction mimicking metachronous metastasis in patients after distal gastrectomy for gastric cancer: a case report and literature review

**DOI:** 10.1186/s40792-020-00955-1

**Published:** 2020-07-29

**Authors:** Haruhiko Okada, Kazutomo Ouchi, Tetsuya Saito, Yuka Takahashi, Masaki Yamada, Naoki Negami, Yasunori Ishido, Sanae Yamazaki, Masahiko Sato

**Affiliations:** 1 Department of Surgery, Saiseikai Kawaguchi General Hospital, 5-11-5 Nisikawaguchi, Kawaguchi, Saitama, 332-8558 Japan; 2 Department of Pathology, Saiseikai Kawaguchi General Hospital, Saitama, Japan

**Keywords:** Sarcoid reaction, Gastric cancer, Spleen, Splenectomy

## Abstract

**Background:**

The occurrence of sarcoid reactions has been recognized in various cancers. The common location for observing these granulomas is mainly the lymph nodes, but a rare occurrence in the spleen has been reported.

Almost all splenic sarcoid reactions associated with gastric cancer have been resected synchronously and diagnosed accidentally, and a rare metachronous occurrence of a sarcoid reaction in the spleen after distal gastrectomy can mimic cancer metastasis. We describe a rare case of a splenic sarcoid reaction recognized in a patient with gastric cancer 6 months after distal gastrectomy.

**Case presentation:**

An 82-year-old man underwent laparoscopic distal gastrectomy for gastric cancer (T3N0M0, stage IIA). Six months after gastrectomy, CT and 18F-fluorodeoxyglucose (FDG)-PET/CT showed the appearance of a splenic mass. We diagnosed solitary splenic metastasis from gastric cancer and performed laparoscopic-assisted splenectomy. His splenic tumor was diagnosed as a sarcoid reaction by histopathological examination.

**Conclusion:**

To our knowledge, this is the first report of a splenic sarcoid reaction recognized 6 months after distal gastrectomy for gastric cancer without any chemotherapy. The splenic sarcoid reaction and cancer metastasis to the spleen were undistinguishable from the CT and FDG-PET/CT findings. The present case and literature review showed that cases of splenic sarcoid reactions associated with gastric cancer can also be accompanied by the occurrence of these granulomas in lymph nodes. When the appearance of a solitary mass is observed in the spleen after resection of primary cancer, it is necessary to consider not only cancer metastasis but also sarcoid reactions. Retrospective histopathological confirmation of the existence of sarcoid reactions in lymph nodes from resected specimens might possibly avoid incorrect diagnosis and intervention.

## Background

Sarcoid reactions characterized by noncaseating epithelioid granulomas without systemic sarcoidosis occasionally occur in various malignant tumors [1]. Sarcoid reactions are mainly observed in the lymph nodes, and splenic sarcoid reactions are rare [2]. In gastric cancer patients, most splenic sarcoid reactions have been recognized accidentally at the preoperative investigation, from intraoperative findings, or during histopathology examinations from gastrectomy and splenectomy specimens [3–10]. The metachronous occurrence of sarcoid reactions in the spleen after gastrectomy is infrequent and mimics cancer metastasis.

Herein, we describe a rare case of a splenic sarcoid reaction mimicking solitary metastasis from gastric cancer identified 6 months after distal gastrectomy.

## Case presentation

**Fig. 1:**
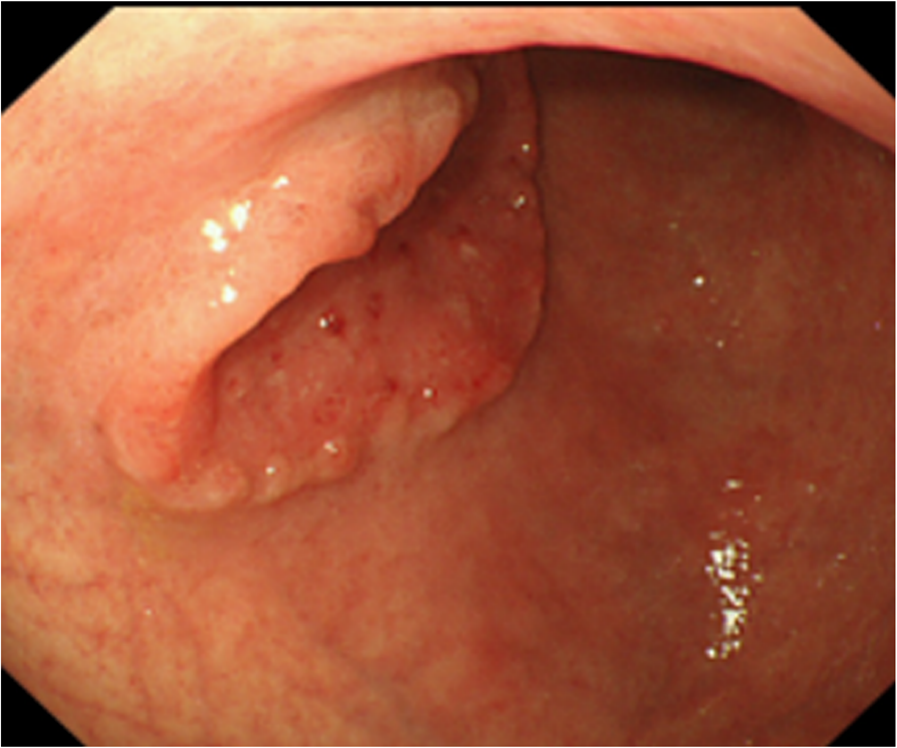
Endoscopy showing type 2 tumor measuring 50 mm at the anterior wall of the lower third of the stomach

The patient was an 82-year-old man diagnosed with gastric cancer (ML, Ant, T3N0M0, cStage IIA, UICC-7) (Fig. 1). Although the preoperative serum carcinoembryonic antigen (CEA) level was as high as 75.9, several imaging studies, including contrast-enhanced computed tomography (CT) and 18F-fluorodeoxyglucose (FDG) positron emission tomography (PET)/CT, showed no metastasis to distant organs or regional lymph nodes. He underwent laparoscopic distal gastrectomy (Billroth-I reconstruction, delta anastomosis) with D2 lymph node dissection. Histopathological examination showed tubular adenocarcinoma (pType2, 50 × 35 mm, tubular adenocarcinoma, well-differentiated type, pT3, INFb, Ly1a, v0, pN0(0/48), pStageIIA).

During the postoperative follow-up, the serum CEA level decreased to within normal limits.

**Fig. 2:**
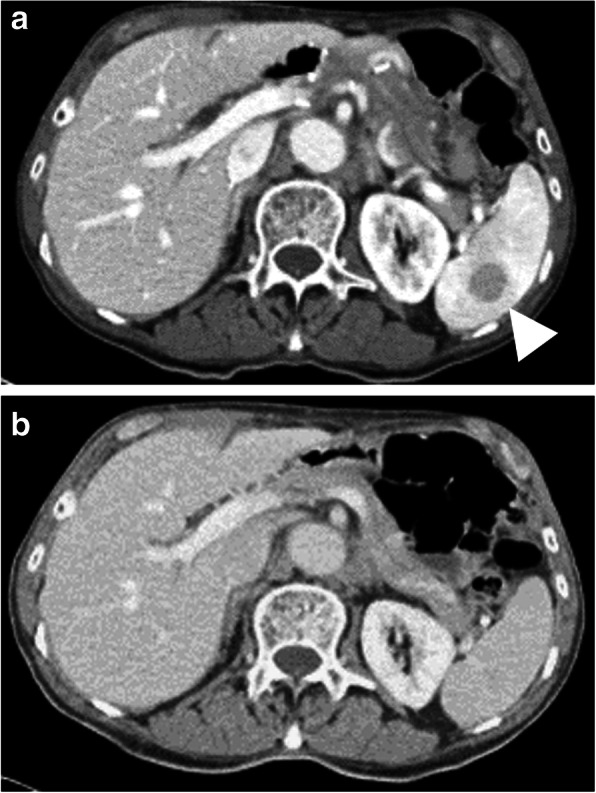
**a** The CT scan 6 months after gastrectomy shows the appearance of a low-density lesion that is 17 mm in diameter at the spleen. **b** Preoperative CT scan shows no lesion at the spleen recognized

**Fig. 3:**
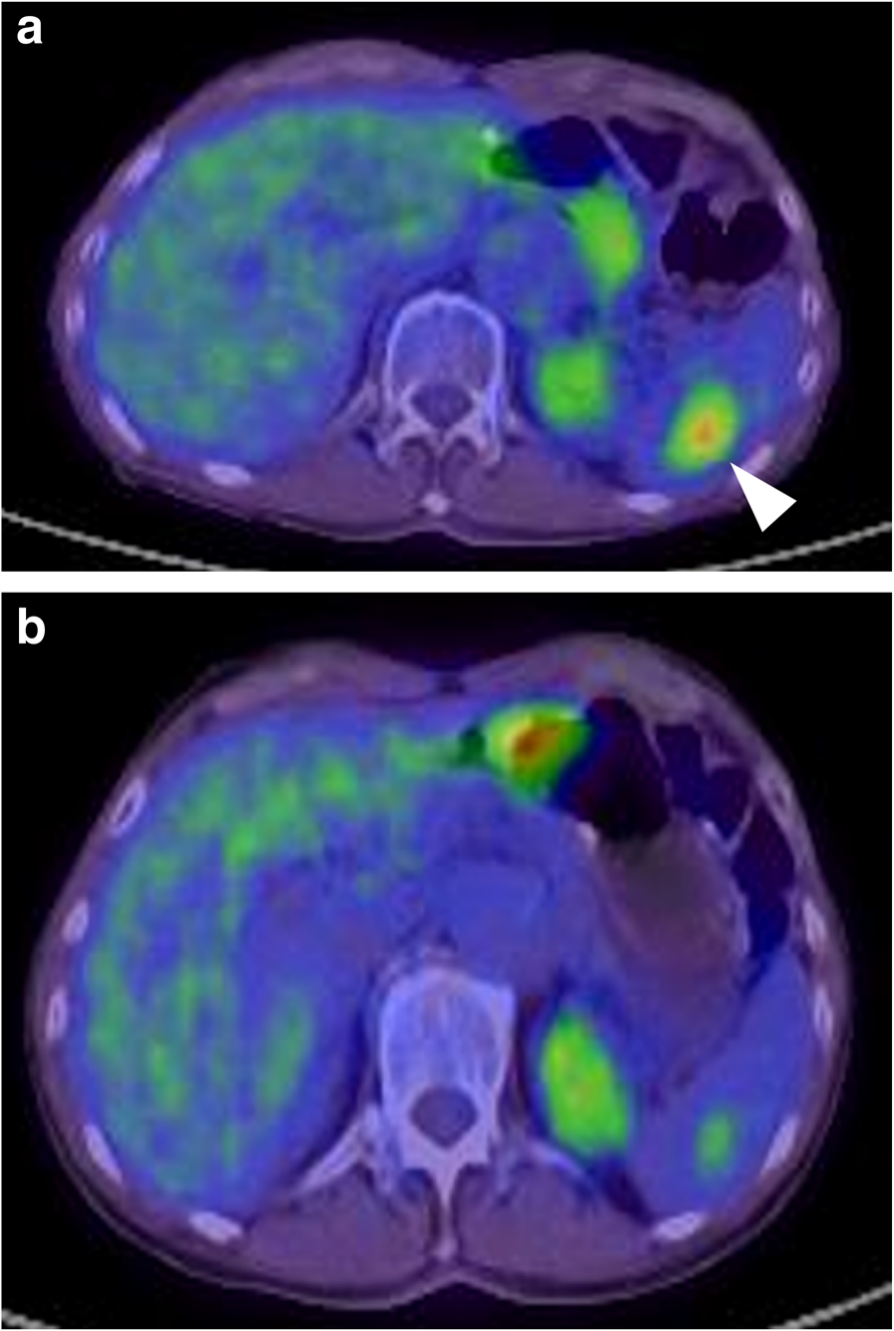
**a** The PET/CT shows intense fluorine-18 fluorodeoxyglucose (18F-FDG) uptake with a maximum standardized uptake value (SUV) of 5.82 (arrowhead) at the spleen. **b** Retrospectively, preoperative PET/CT shows very faint uptake (maximum SUV, 4.76) at the spleen

Contrast-enhanced CT 6 months postoperatively showed a small low-density lesion, 17 mm in diameter, in the spleen (Fig. 2). Whole-body PET/CT showed high FDG uptake at the site of the mass (maximum standardized uptake value (SUV), 5.82) (Fig. 3a).

**Fig. 4:**
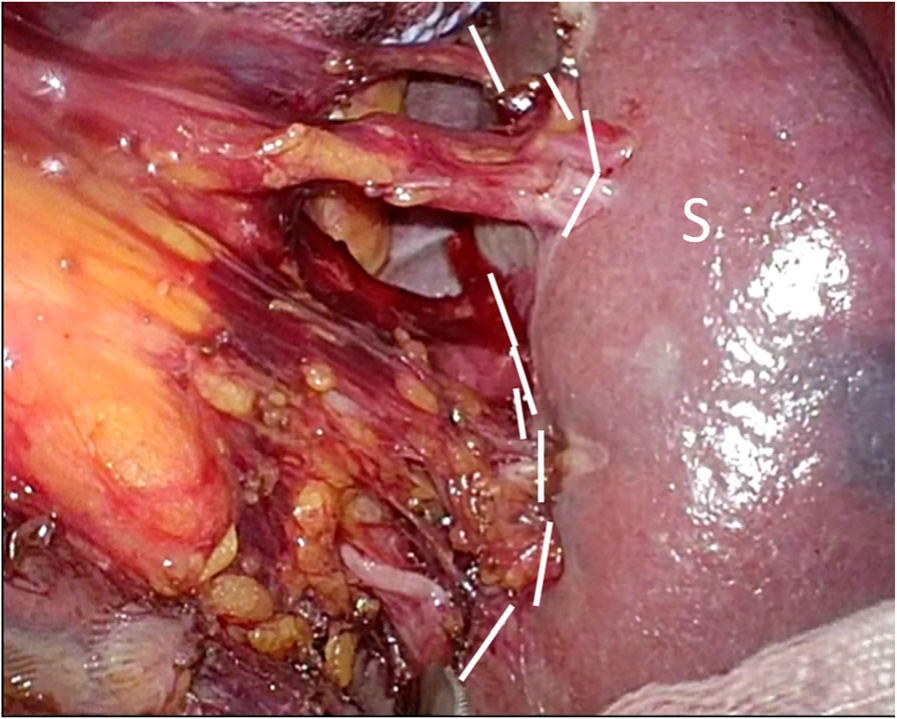
Intraoperative image showing the dissected line (broken line) just at the point of the splenic hilum, and the short gastric vessels in the gastrosplenic ligament were preserved as much as possible to supply the remnant stomach. S, spleen

**Fig. 5:**
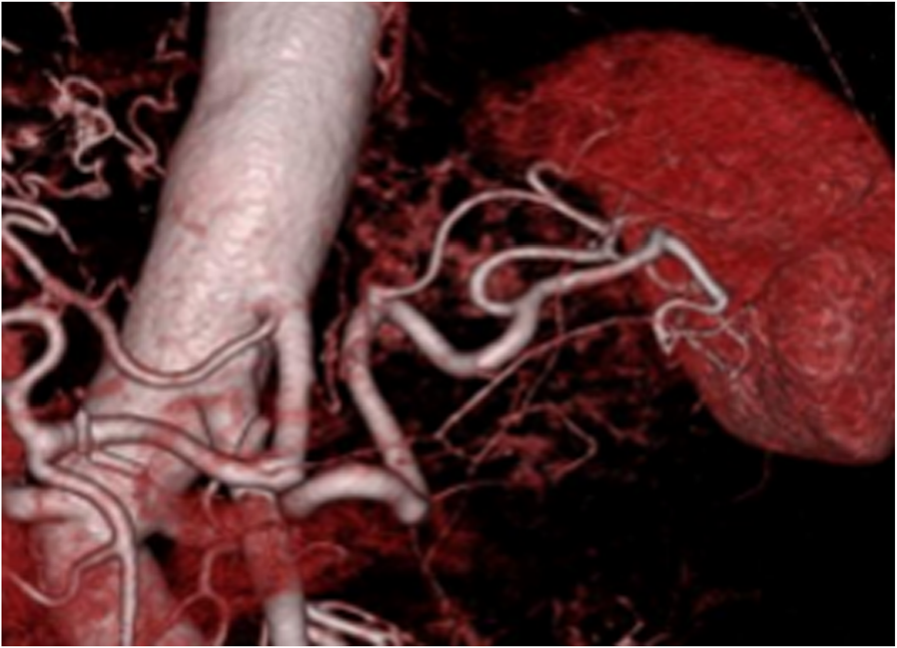
3D-CT angiography to evaluate the formation of the vessels at the hilum of the spleen

Extrasplenic metastasis or dissemination was not suspected. Retrospectively, very faint uptake was found on preoperative PET/CT (maximum SUV, 4.76) (Fig. 3b), which increased in size. The mass was suspected to be solitary metastasis of primary gastric cancer. Metastasis was isolated, and we thought it was resectable because there was no other metastasis. Finally, the patient opted for radical splenectomy. The patient was admitted to our hospital for surgery and underwent laparoscopic-assisted splenectomy. Briefly, the splenic vessels were dissected just at the point of the splenic hilum, and the short gastric vessels in the gastrosplenic ligament were preserved as much as possible to supply the remnant stomach (Fig. 4). The most concerning problem associated with splenectomy was the ischemic complications of the remnant stomach with abrupt sacrifice of the short gastric vessels. We evaluated the formation of these vessels at the hilum of the spleen using 3D-CT angiography (Fig. 5), abdominal angiography, and previous surgical videos of this patient preoperatively.

The postoperative course was uneventful, and no ischemic complications of the remnant stomach developed with evaluation using contrast-enhanced CT on postoperative day 1 and esophagogastroduodenoscopy (EGD) on postoperative day 2. The patient was discharged on postoperative day 10.

**Fig. 6:**
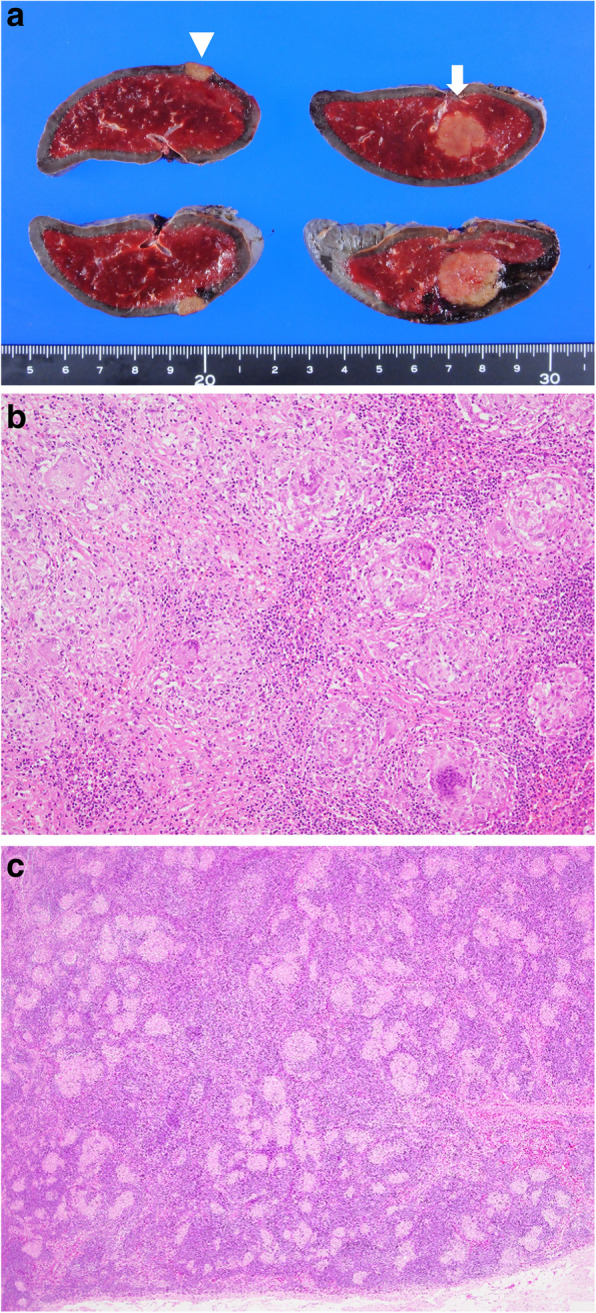
**a** Operative specimen showing two splenic tumors measuring 20 mm (arrow) and 7 mm (arrowhead). **b** Histopathological findings of the spleen showing epithelioid cell granulomas without caseous necrosis and no evidence of malignant cells. **c** The retrospective histopathological findings of the regional lymph nodes show noncaseous epithelioid granulomas (sarcoid reaction)

The operative specimen revealed a splenic tumor measuring 20 mm in diameter and another unexpected tumor measuring 7 mm in diameter (Fig. 6a). These splenic tumors showed noncaseating epithelioid cell granulomas and no evidence of malignant cells (Fig. 6b).

Then, we performed a retrospective histopathological examination of the primary gastric cancer with regional lymph nodes and observed the same granulomas as in the sarcoid reactions in a portion of the regional lymph nodes (station nos. 7, 8a, 9, 11p, 12a. 11/48) (Fig. 6c).

There were no findings indicating systemic sarcoidosis, including skin or ocular lesions; the levels of serum angiotensin-converting enzyme (s-ACE) were within the normal range; and there was no accumulation observed with Gallium-67 scintigraphy.

We made a diagnosis of a splenic sarcoid reaction associated with advanced gastric cancer.

At the 24-month follow-up, the patient was well and healthy without any evidence of gastric cancer recurrence.

## Discussion

Sarcoidosis is a granulomatous disease of unknown etiology characterized by compartmentalization of CD4 T helper 1 (TH1) cells and activated monocyte/macrophages in the organs involved [11]. The diagnosis of sarcoidosis requires the subjective criteria of a clinical presentation, such as dyspnea, low-grade fever, weight loss, skin rashes, and vision changes. The same noncaseating epithelioid cell granulomatous appearance may occur on occasion in lymph nodes draining a region housing a malignant tumor, in the primary tumor itself and, rarely, in nonregional tissues such as the spleen or liver, called a sarcoid reaction [1]. Sarcoid reactions associated with malignant tumors lack the clinical symptoms of sarcoidosis. Brinker demonstrated that sarcoid reactions caused by antigenic factors were derived from the formation of epithelioid cell granulomas and that they may be a marker of an immunologically mediated antitumor response of macrophages activated by T lymphocytes [1].

Recently, the use of immune checkpoint inhibitors, which have been expanded for use in several cancers, has been reported for the occurrence of sarcoid reactions called drug-induced sarcoid reactions [11–14]. T cell proliferation and increased expression of T helper (Th) 1-associated markers can potentially induce these drug-induced sarcoid reactions [12].

There have been no definitive reports about the relationships among pathogeneses of systemic sarcoidosis, sarcoid reactions associated with malignancy, and drug-induced sarcoid reactions. Favorable outcomes of advanced gastric cancer patients with sarcoid reactions and the possibility of the active involvement of the host immune system against tumors have been reported for some time [4, 15]. Chopra et al. reported the necessity to distinguish sarcoid reactions associated with malignancy from drug-induced sarcoid reactions [12], but the investigation into the pathophysiologic mechanisms of immunotherapy-related sarcoid reactions might contribute to elucidating the immunopathologic mechanisms of sarcoid reactions associated with cancers.

Kojima et al. reviewed 100 gastric cancer patients and reported that the frequency of a sarcoid reaction was 5% in the spleen and 13% in the regional lymph nodes [3].

Literature searches were executed for related reports from which we could obtain clinicopathological information published between January 1997 and April 2020 in Japan Medical Abstracts Society WEB (the largest Japanese medical database) and from the earliest possible date to April 2020 in PubMed using the keywords “gastric cancer,” “spleen,” and “sarcoid reaction,” which comprised 12 patients, with the present case total of 13 patients (12 with splenic sarcoid reactions and 1 with sarcoidosis) showing in Table 1 [3–10]. In our analysis of these cases, categorization by age showed a predominance for older populations (11 of 13 patients were older than 65 years). The histological type varied and included signet ring cell carcinoma, tubular adenocarcinoma, and small cell carcinoma. Categorization by TMN indicated a predominance of advanced stages (12 of 13 patients). Eleven of 13 patients underwent total gastrectomy and simultaneous splenectomy, and one patient underwent distal gastrectomy for gastric cancer and fine needle aspiration (FNA) for splenic tumors with already diagnosed sarcoidosis. In terms of the categorization by the time of identifying a splenic mass, 4 of 13 patients were identified by preoperative imaging (CT and PET/CT), 1 patient by intraoperative findings, and 7 patients by postoperative surgical specimens. All 3 patients who underwent PET/CT were diagnosed with splenic metastasis from gastric cancer with high FDG uptake preoperatively. All 12 splenic sarcoid reaction patients except one sarcoidosis patient also had occurrence in the lymph nodes. In terms of the categorization by a form of splenic sarcoid reaction with information available, 7 of 8 patients showed multiple reactions (1 case of a solitary reaction by CT and multiple reactions by surgical specimens). The present case showed solitary lesions by CT and PET/CT and two lesions by surgical specimens.

**Table 1:** Summary of patients with gastric cancer and splenic sarcoid reaction/sarcoidosis

No	Year	Author	Age	Sex	Diagnosis	Form of splenic SR	Time of diagnosis with splenic mass	PET/CT	Location of SR	Location of GC	Histological type	Operation	TNM
1	1997	Kojima et al. [3]	67	M	SR	NA	Postoperative (ss)		LN + S	NA	Diffuse	TG + S	T2N1
2			72	M	SR	NA	Postoperative (ss)		LN + S	NA	Diffuse	TG + S	T3N1
3			73	F	SR	NA	Postoperative (ss)		LN + S	NA	Diffuse	TG + S	T3N1
4			74	M	SR	NA	Postoperative (ss)		LN + S	NA	Intestinal	TG + S	T4N2
5			77	M	SR	NA	Postoperative (ss)		LN + S	NA	Intestinal	TG + S	T3N2
6	1999	Shigematsu et al. [4]	66	M	SR	Multiple	Intraoperative findings		LN + S	UML	Well to poor	TG + S + C	T2N1
7	1999	Igarashi et al. [5]	69	F	SR	Multiple	Postoperative (ss)		LN + S	UE	Small cell carcinoma	TG + PS	T4Nx
8	2000	Kiyasu et al. [6]	72	M	SR	Multiple	Postoperative (ss)		LN + S	UE	Mod	TG + S	T3N3
9	2008	Gondou et al. [7]	58	F	SR	Multiple	Preoperative CT		LN + S	U	Poor	TG + S + C	T2N2
10	2010	Ohta et al. [8]	53	F	SR	Multiple	Preoperative CT		LN + S	U	Poor	TG + S + C	T1bN0
11	2011	Mikami et al. [9]	70	F	Sarcoidosis	Multiple	Preoperative CT and PET/CT	+	S	ML	Poor	DG + FNA	T3N3a
12	2012	Konishi et al. [10]	69	F	SR	Solitary by image, multiple by ss	Preoperative PET/CT	+	Stomach + LN + S	M	Sig	TG + S	T2N1
13		Our case	82	M	SR	Solitary by image, two by ss	Postoperative (6 months) (CT and PET/CT)	+	LN + S	ML	Well	DG → S	T3N0

*SR* sarcoid reaction, *NA* not available, *ss* surgical specimen, *LN + S* lymph node and spleen, *S* spleen, *E* esophagus, *U* upper third of stomach, *M* middle third of stomach, *L* lower third of stomach, *well* well-differentiated adenocarcinoma, *poor* poorly differentiated adenocarcinoma, *sig* signet ring cell carcinoma, *TG* total gastrectomy, *S* splenectomy, *PS* distal pancreatectomy + splenectomy, *C* cholecystectomy, *DG* distal gastrectomy, *FNA* fine needle aspiration

All reported patients with splenic sarcoid reactions and gastric cancer except our present case underwent total gastrectomy and simultaneous splenectomy and were then pathologically diagnosed with sarcoid reactions. There have been no reports of a case of a splenic sarcoid reaction after distal gastrectomy in a patient with gastric cancer without any adjuvant therapy.

Appearance of a splenic mass during cancer treatment generally suggests metastasis from primary cancer. The frequency of splenic metastasis from gastric cancer has been reported as 4.1–6.9% [16], which is almost the same as that of splenic sarcoid reactions (5%) [3]. The treatment strategies used to isolate splenic metastasis from gastric cancer are still controversial. Isolated splenic metastasis is very rare, and once splenic metastasis occurs, it is usually accompanied by multiorgan metastasis and dissemination because of the malignant nature of gastric cancer. Therefore, systemic chemotherapy is generally a consideration for splenic metastasis as a systemic disease.

Yoshizawa et al. reviewed 19 cases of isolated splenic metastasis from gastric cancer with splenectomy [16]. He reported 8 cases among the 14 metachronous metastasis cases that demonstrated relapse-free survival for longer than 12 months, and the prognosis of isolated metachronous splenic metastasis from gastric cancer may be favorable.

The present case was an elderly patient diagnosed with splenic metastasis from gastric cancer. Systemic chemotherapy was difficult because of his advanced age, and finally, he opted for radical resection of the splenic tumor.

He underwent splenectomy after a diagnosis of isolated gastric cancer metastasis to the spleen and histopathological examination of noncaseating epithelioid cell granulomas. Then, retrospective confirmation of these granulomas in lymph nodes in the previously resected specimen led to the diagnosis of a splenic sarcoid reaction.

Nakamura et al. reported that because sarcoid reactions in resected lymph nodes represent a relatively ordinary phenomenon in gastric cancer, pathologists may not always report these small granulomas or may downplay them [15].

For patients with a history of distal gastrectomy, the decision of splenectomy should be made cautiously due to ischemic complications of the remnant stomach [17, 18]. Arterial blood flow to the remnant stomach after distal gastrectomy consists of the short gastric artery, the posterior gastric artery, and the recurrent branches of the left inferior artery in some cases. Takahashi et al. reported that a relatively long interval after distal gastrectomy might fertilize the collateral blood supply and the intramural network of the mucosal and submucosal plexuses and stabilize the blood supply to the remnant stomach [17]. The interval of the present case was less than 1 year after distal gastrectomy, which is a disadvantage for forming potent blood supply to the remnant stomach. Intraoperative injury of these vessels might lead to the development of ischemic complications involving the remnant stomach. Splenectomy with total gastrectomy can be fatal, especially in elderly patients. In the present case, we made efforts to review the formation of the vessels at the hilum of the spleen with 3D-CT angiography, abdominal angiography, and previous surgical videos with gastrectomy and to preserve the short gastric arteries as much as possible to avoid the development of remnant stomach ischemia. Recently, the efficacy of indocyanine green (ICG) fluorescence angiography has been reported to be helpful for evaluating blood flow in the remnant stomach [17].

Splenectomy also involves the risk of overwhelming postsplenectomy infection (OPSI) as a critical life-threatening complication.

Less invasive investigations, such as FNA, are generally thought to be difficult because of the risk of intra-abdominal dissemination of the tumor in cases strong suspected of cancer metastasis [16]. But in a case of gastric cancer with a splenic mass which could not diagnose to cancer metastasis and including the possibility of a sarcoid reaction, it might need not to hesitate to perform intraoperative needle biopsy with preparing splenectomy.

The main problem is the difficulty of distinguishing cancer metastasis from sarcoid reactions with imaging modalities such as contrast-enhanced CT or FDG-PET [19, 20].

Cancer metastasis requires treatment such as surgical intervention or systemic chemotherapy, but these treatments are unnecessary in sarcoid reactions [20].

As Kojima et al. and Kiyasu et al. reported [3, 6], the present case and literature review showed that cases of splenic sarcoid reactions also occur in the lymph nodes. If the appearance of splenic tumors after gastrectomy in gastric cancer patients is recognized, it is necessary to suspect not only cancer metastasis but also the possibility of a sarcoid reaction. Then, retrospective histopathological examination of the lymph nodes to confirm the existence of a sarcoid reaction would be helpful for optimal treatment.

## Conclusion

We present a rare case of a splenic sarcoid reaction recognized 6 months after distal gastrectomy for gastric cancer without any chemotherapy. Splenic sarcoid reactions and cancer metastasis to the spleen are undistinguishable from CT and FDG-PET findings. Our case and literature review showed that cases of splenic sarcoid reactions also occur in the lymph nodes. When the appearance of a solitary mass is observed in the spleen after gastrectomy in gastric cancer patients, it is necessary to consider not only cancer metastasis but also sarcoid reactions. Retrospective histopathological confirmation of the existence of a sarcoid reaction in the lymph nodes from the resected specimen would be helpful for optimal diagnosis.

## Data Availability

Not applicable

## Abbreviations

SR: Sarcoid reaction
GC: Gastric cancer
CT: Computed tomography
18F-FDG: Fluorine-18 fluorodeoxyglucose
PET: Positron emission tomography
CEA: Carcinoembryonic antigen
FNA: Fine needle aspiration
SUV: Standardized uptake value
